# A ‘devil’ of a problem

**DOI:** 10.7554/eLife.39976

**Published:** 2018-08-14

**Authors:** Victoria L Hansen, Robert D Miller

**Affiliations:** 1 Animal Biosciences and Biotechnology LaboratoryUnited States Department of Agriculture/Agricultural Research ServiceBeltsvilleUnited States; 2 Center for Evolutionary & Theoretical Immunology, Department of BiologyUniversity of New MexicoAlbuquerqueUnited States

**Keywords:** *Sarcophilus harrisii*, MHC, contagious cancer, immune escape, MHC-1, Tasmanian devil, Other

## Abstract

The discovery of a second facial tumor disease in the Tasmanian devil has provided insights into the emergence of contagious cancers.

**Related research article** Caldwell A, Coleby R, Tovar C, Stammnitz MR, Kwon YM, Tringides M, Murchison EP, Skjødt K, Thomas GJ, Elliot T, Woods G, Siddle HV. 2018. The newly-arisen Devil facial tumour disease 2 (DFT2) reveals a mechanism for the emergence of a contagious cancer. *eLife* **7**:e35314. doi: 10.7554/eLife.35314

Times are tough for the Tasmanian devil – nature's largest carnivorous marsupial. Over the past two decades, a contagious form of cancer called devil facial tumor disease has been wreaking havoc on the Tasmanian devil and may even be driving it to extinction (McCallum and Jones, 2006).

Cancers are not usually contagious, but devil facial tumor disease (DFTD) is, with tumor cells being passed between individual devils, much like viruses or bacteria. The disease is transmitted when devils bite each other during territorial disputes or courtship, making them most susceptible as they reach sexual maturity. Once the tumors are evident, the life expectancy of a devil is less than six months (Hawkins et al., 2006). One consequence of DFTD is that devils are reaching sexual maturity at a younger age, illustrating the strong selective pressure the disease is putting on this species (Jones et al., 2008).

So far, transmissible cancers have been found in just a few species besides the Tasmanian devil – notably dogs and soft-shell clams (Metzger et al., 2015; Murgia et al., 2006). DFTD is remarkable in that two – apparently independent – cancers have arisen in wild devil populations over a relatively short period of time. Tasmanian devils with tumors and lesions in the face caused by the highly contagious DFT1 were first sighted in 1996 (Hawkins et al., 2006); DFT2, which is thought to be less pathogenic, was discovered in 2014 (Pye et al., 2016).

One transmissible cancer in a species may be unfortunate, but two suggest that there is something more going on. Now, in eLife, Hannah Siddle and colleagues – including Alison Caldwell as first author – report that DFT2 may be evolving tactics to evade the immune system that are similar to those used by DFT1 (Caldwell et al., 2018).

The ability to distinguish self from non-self molecules is at the core of immunology, and the underlying mechanisms are generally well established. In the immune system of vertebrates, a cluster of genes called the major histocompatibility complex (MHC) enables the body to recognize and destroy abnormal tissues (such as infected or tumorous tissues) and foreign tissues (like organ transplants; see Schwartz, 1985 for a review).

One group of MHC genes, the class I (MHC-I), encodes molecules that play an important role in this process. Most cells display MHC-I molecules on their surfaces, and these molecules present peptides which reveal if the cell is self or not: if a peptide indicating a mutated-self of pathogenic cell is detected, a specific type of immune cell, called cytotoxic T cell, is alerted and sent to destroy the cell.

However, cancer cells or cells infected with a virus, often escape death at the hands of these T cells by losing their MHC-I molecules, which is one of the mechanisms employed by DFT1 (Siddle et al., 2013). Consequently, when MHC-I expression is reduced or lost, T cells are unable to see the tumor or infected cell. By studying DFT2 cancer cells taken from biopsies or grown in the laboratory, Caldwell et al. – who are based at the universities of Southampton, Tasmania, Cambridge and Southern Denmark – discovered that DFT2 appears to use a similar tactic (Figure 1). While it still has MHC-Is, DFT2 seems to be slowly evolving to express fewer of them on its surface – potentially becoming as contagious as DFT1.

**Figure 1.**
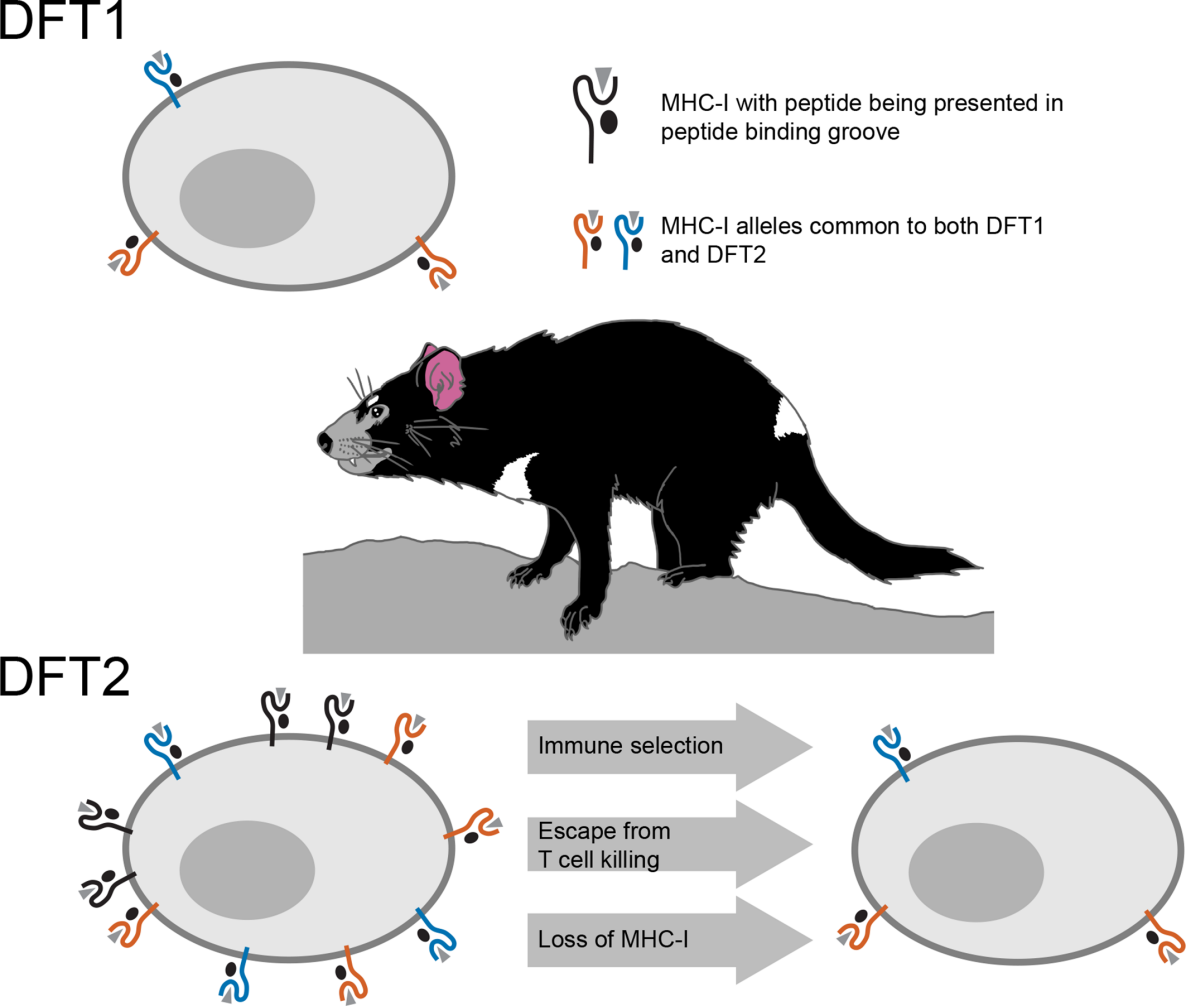
Schematic illustrating the mechanisms underlying devil facial tumor disease. The immune system identifies cells that should not be in an organism based on foreign or abnormal peptides (grey triangles) presented in the binding groove of MHC-I molecules on the surface of the cell. DFT1 cells (top) avoid the immune system of the Tasmanian devil by losing expression of their MHC-I molecules (orange and blue). DFT2 cells (bottom left) appear to be developing a similar trick, but still express a number of MHC-I molecules (shown in black) that can be recognized by the immune system. However, Caldwell et al. found that DFT2 cells are starting to lose MHC-I molecules (bottom right), which suggests that this form of tumor could soon become as pathogenic as DFT1. DFT1 and DFT2 also appear to share gene alleles of MHC-I genes (orange and blue), suggesting there may be something about these variants associated with the emergence of a contagious tumor.

Usually, the MHC differs between the individuals of a species due to genetic variation. MHC molecules encoded by different gene variants bind and present different sets of peptides. This enhances the killing abilities of T cells by providing a diversity of peptides for T cells to recognize and respond against. This also contributes to the need for genetic matches between an organ donor and a recipient. The MHC-I molecules of DFT2, however, are genetically similar among Tasmanian devil populations, and are therefore less likely to be seen as foreign by T-cells. Caldwell et al. found that DFT1 and DFT2 share MHC-I genes, suggesting that something about these particular variants may be conducive to avoiding the devil immune system.

The emergence of DFT2 provides new insights into the early evolution of transmissible tumors. Nevertheless, many questions persist. It remains to be seen whether these tumors are more common in nature than we assume. If they were indeed so rare, why did they arise twice in the same species? Could specific features of their immune system or their (loss of) environment make the Tasmanian devil more susceptible to the emergence of transmissible tumors?

However, an increasing number of animal species are experiencing both a reduction in number (and, therefore, a reduction in genetic diversity) and a loss of natural habitats. If the emergence of transmissible tumors is dictated by a set of rules rather than plain bad luck, we may discover more cases of contagious cancers that take advantage of common MHC-I alleles. Tasmanian devils and other endangered species may be particularly vulnerable to cancers exploiting loop-holes in their immune systems.
